# Hydrolytic degradation behaviour of electrospun poly(ɛ-caprolactone) filaments for biological tissue repair

**DOI:** 10.1016/j.jmbbm.2025.107308

**Published:** 2026-03

**Authors:** Thales Zanetti Ferreira, Huanming Chen, Kaili Chen, Pierre-Alexis Mouthuy, Laurence Brassart

**Affiliations:** a Department of Engineering Science, University of Oxford, Oxford OX1 3PJ, United Kingdom; b Botnar Institute of Musculoskeletal Sciences, Nuffield Department of Orthopedics, Rheumatology and Musculoskeletal Sciences, University of Oxford, Oxford OX3 7LD, United Kingdom

**Keywords:** Electrospinning, Biomedical fibres, PCL, Mechanical characterisation

## Abstract

Electrospun polymeric fibres are promising materials for biomedical applications, owing to their biocompatibility, biodegradability, and ability to be assembled into a non-woven fibrous mesh. In particular, continuous filaments can be produced and subsequently assembled into multi-filament braided structures for ligament and tendon tissue repair. In these applications, characterising the evolution of the mechanical properties of the filament as it degrades is of primary importance. The role of applied mechanical loads during the degradation process also needs to be understood. In this study, we characterised the hydrolytic degradation behaviour of pre-stretched electrospun filaments made of poly(ɛ- caprolactone) (PCL) in buffer saline solution at 45 °C for up to 5 weeks, considering both non-loaded and loaded conditions. We show that PCL filaments degrade significantly over this relatively short time period, with non-loaded specimens showing a 21 % reduction in molecular weight after 5 weeks of exposure. Tensile loads applied during degradation further accelerate the degradation rate, with filaments subjected to a 25 g load showing a 33 % reduction in molecular weight over the same time period. Applied loads also impact the mechanical properties of the degraded specimens, causing an increase in elastic modulus and strength but a sharp decrease in elongation at break with exposure time. Our findings have implications for the design of PCL electrospun constructs in load bearing biomedical applications.

## Highlights

• Significant hydrolytic degradation observed after 5 weeks in PBS at 45 °C.
• Tensile loads accelerate degradation of electrospun PCL filaments.
• Loads applied during degradation increase the elastic modulus and strength but reduce ductility.
• Effect of loads on degradation discussed based on microstructural evolution.

## Graphical abstract

**Figure:**
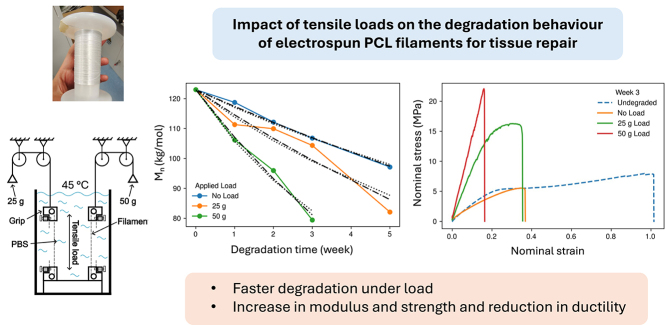

## 1 Introduction

Electrospinning (ES) is an attractive technique for the processing of biomaterials, owing to its ability to produce lightweight, fibrous structures that closely mimic the extracellular matrix. Applications of ES constructs for biomedical applications include scaffolds for tissue engineering, wound healing devices, and drug delivery systems (Mouthuy and Ye, 2011, Ibrahim and Klingner, 2020, Guo et al., 2022). Various polymers have been successfully electrospun to date, including biodegradable polymers such as poly(lactic acid) (PLA), poly(lactic-co-glycolic acid) (PLGA) and poly(ɛ-caprolactone) (PCL), as well as natural polymers such as collagen and gelatin (Mouthuy and Ye, 2011).

In recent years, continuous ES filaments consisting of aligned microfibres have been proposed as a promising biomaterial for ligament and tendon repair (Mouthuy et al., 2015, Savić et al., 2021). Continuous ES filaments can be stretched and processed into braided yarns, which can in turn be used to create biomedical implants with tailored mechanical properties. In our recent work, we have characterised the mechanical properties of PCL ES filaments, focusing on their large deformation, rate-dependent response (Zanetti Ferreira et al., 2025). Another recent study investigated the microstructural changes within the microfibres of ES PCL filaments during stretching (Chandler et al., 2025). However, the degradation behaviour of this material has not yet been investigated.

PCL is a semi-crystalline polymer with low glass transition temperature ($T_{g}\approx -60\;{}^{\circ}C$) and low melting temperature ($T_{m}\approx 60\;{}^{\circ}C$). It has been extensively used for biomedical applications, owing to its biodegradability, biocompatibility and ease of processing (Woodruff and Hutmacher, 2010, Bartnikowski et al., 2019). In physiological conditions, the degradation of PCL primarily occurs by bulk erosion through hydrolytic cleavage of ester groups. Compared to other polyesters, it exhibits significantly slower degradation (approximately 2–4 years to full breakdown) due to the presence of five hydrophobic −CH${}_{2}$ moieties in its repeating units, which increases its resistance to hydrolysis (Dwivedi et al., 2020). The slow degradation of PCL is also due to its high degree of crystallinity (up to 70% depending on the molecular weight, Sisson et al., 2013), since the dense packing of polymer chains in the crystalline regions hinders water penetration, resulting in a much slower degradation of the crystalline domains (Hakkarainen, 2002, Jenkins and Harrison, 2008).

Over the past decades, numerous studies have investigated the degradation of PCL in both physiological (Pitt et al., 1981, Tsuji and Ikada, 1998, Yavuz et al., 2002, Coombes et al., 2004, Lam et al., 2009, Domingos et al., 2010) and accelerated degradation conditions (Lam et al., 2008, Little et al., 2009, Chung et al., 2012, Sailema-Palate et al., 2016). However, studies focusing on the degradation behaviour of PCL ES fibres remain limited (Bölgen et al., 2005, Johnson et al., 2009, Vieira et al., 2011, Limbert et al., 2016, Bazgir et al., 2021). The degradation mechanisms and kinetics of ES networks are expected to differ from those of bulk PCL, primarily due to differences in surface area-to-volume ratio, as well as changes in hydrophobicity and crystallinity induced by the electrospinning process (Dong et al., 2009, Cipitria et al., 2011). For example, Bölgen et al. (2005) observed a greater degradation rate in ES construct with thinner fibres, which was attributed to their higher surface-to-volume ratio, enabling higher water penetration.

Another important factor that could impact the degradation behaviour of ES constructs is the presence of applied loads. To date, there have been only limited attempts at characterising the degradation behaviour of polyesters degrading under applied loads, and findings have been somewhat contradictory. For example, Deng et al. (2005) investigated the degradation response of PLGA multifilament braids under tensile loads, but found that the loads did not affect the degradation rate (measured by the reduction in molecular weight) significantly. Samanta et al. (2025) reported that applied tensile loads reduced the degradation rate of PLGA specimens, and further impacted their degradation mechanism. In contrast, other studies found that tensile loads accelerate the degradation of PLGA electrospun scaffolds (Li et al., 2010) and PLGA membranes (Guo et al., 2016). Recently, Chen et al. (2026) found that both tensile and compressive loads accelerate the degradation of PLA below a certain molecular weight threshold. However, these materials are typically in their glassy state upon implantation, and therefore the mechanisms for force-assisted degradation may be different than in PCL. To the best of our knowledge, the effect of tensile loads on the hydrolytic degradation of PCL ES constructs has not been investigated before.

The objective of this study is to investigate the effect of mechanical loads on the hydrolytic degradation behaviour of PCL ES filaments. Accelerated degradation tests were conducted in Phosphate Buffer Saline (PBS) solution at 45 °C for up to 5 weeks, and filaments were subjected to a tensile weight during degradation using a custom-designed rig. Characterisation of the degraded filaments include measurements of the molecular weight by Gel Permeation Chromatography (GPC), thermal analysis by Differential Scanning Calorimetry (DSC), morphological evaluation by Scanning Electron Microscopy (SEM), and uniaxial tensile tests. Our results show a significant effect of mechanical loads on the degradation rate of the filaments, accompanied by reduction in strain at break but an increase in modulus and strength. Results are discussed in light of possible mechanisms for microstructure evolution in filaments degrading under load.

## 2 Experimental methods

### 2.1 Polymer solution preparation

Electrospinning solutions were prepared by dissolving PCL with initial weight average molecular weight of approximately $M_{w}=175$ kg mol^−1^ according to the manufacturer (Ashland Specialities, Ireland) in 1,1,1,3,3,3-hexafluoroisopropanol (HFIP) (Apollo Scientific Ltd, UK). The polymer was dissolved at a 10% weight-to-volume (w/v) ratio by dissolving 10 g of PCL in 100 mL of HFIP. The solutions were agitated at room temperature using a roller mixer (Stuart SRT9D, UK) set to 21 rpm for a minimum of 24 h to ensure complete dissolution.

### 2.2 Electrospinning

**Fig. 1:**
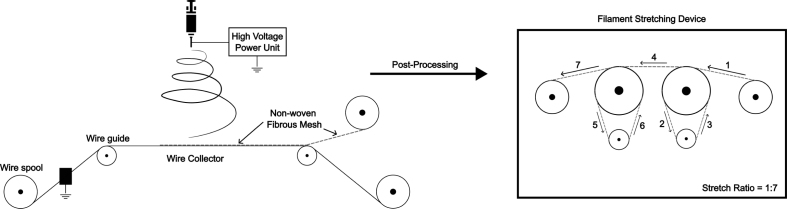
Schematic of the novel electrospinning collection technique. The method consists of electrospinning PCL fibres onto a stainless steel wire progressing at a speed of 0.4 mm s^−1^. The electrospun mesh is separated from the wire and collected in the form of a continuous filament.

Electrospun filaments were fabricated by depositing a continuous fibre onto a thin guided wire, forming a dense and narrow mesh that could be detached as a long, continuous thread, as illustrated in Fig. 1. A detailed description of the production method is provided by Mouthuy et al. (2015). The electrospinning process utilised a single-nozzle setup with a continuous metallic wire (100μm diameter) (Goodfellow, UK), an SL30P30/230 high-voltage power supply (30 kV) (Spellman, UK), and a syringe pump (World Precision Instruments Limited, US). The metallic wire was cleaned with 70% ethanol prior to setup. Electrospinning was conducted within a glove box under constant airflow. The room temperature was maintained at 22±3°C and the relative humidity at 40±3%. The polymer solution was delivered at a rate of 1 mL h^−1^, with the nozzle tip positioned 20 cm from the wire and an applied voltage between 7 kV and 10.5 kV. The wire moved perpendicularly to the nozzle tip at a speed of 0.4 mm s^−1^. The resulting product was a metallic wire coated with a continuous ES mesh. Upon exiting the glovebox, the ES filament was detached from the wire and automatically collected on a rotating spool. Upon completion of electrospinning, spools were placed in grip-sealed bags and stored in a vacuum desiccator at room temperature to remove residual solvent and moisture, ensuring that the filaments remained dry. Filaments were stored under these conditions for periods of up to three months before post-processing stretching.

Dry ES continuous filaments were stretched to an average of seven times their original length using an industrial drawing machine (Retech, Switzerland). Initially, a spool containing 5 m of unstretched material was loaded onto the drawing machine. The filament was then fed through a series of bollards, incrementally stretching it to produce a continuous filament approximately 35 m in length. An illustration of this stretching process is provided in Fig. 1. Pre-stretching of the filaments serves two primary purposes: (i) it increases the stiffness of the filaments and reduces the risk of plastic deformation after implantation, and (ii) it promotes alignment of the fibres in the primary loading direction, mimicking the structure of native fibrous tissue such as tendons and ligaments (Mouthuy et al., 2015, Zanetti Ferreira et al., 2025, Chandler et al., 2025). Pre-stretched ES filament spools were stored in a desiccator to prevent environmental degradation and mechanical damage.

### 2.3 Degradation tests

ES filaments were degraded in PBS (Merck, UK) solution with pH 7.4 at constant temperature of 45 °C up to 5 weeks, and under different applied loads (0, 25, and 50 g). The temperature of 45 °C was identified as the upper limit for accelerated degradation without altering the fundamental degradation mechanism. This temperature is lower than the main melting temperature of PCL (around 60 °C). It also corresponds to the onset of the endothermic peak in DSC tests of pre-stretched filaments (Chandler et al., 2025), indicating a destabilisation of the crystalline structure and which could impact the hydrolytic degradation mechanism. In addition, Chung et al. (2012) showed that the temperature-dependence of the hydrolysis reaction kinetics of PCL-based electrospun scaffolds was well described by an Arrhenius relation, based on degradation tests at three temperatures (25 °C, 37 °C and 45 °C) and pH of 7.4. These results suggest that the degradation mechanism does not change within this temperature range. The applied loads were selected to be representative of the service load on individual filaments when used as braided implants within the knee. Filaments degraded under no load served as controls.

**Fig. 2:**
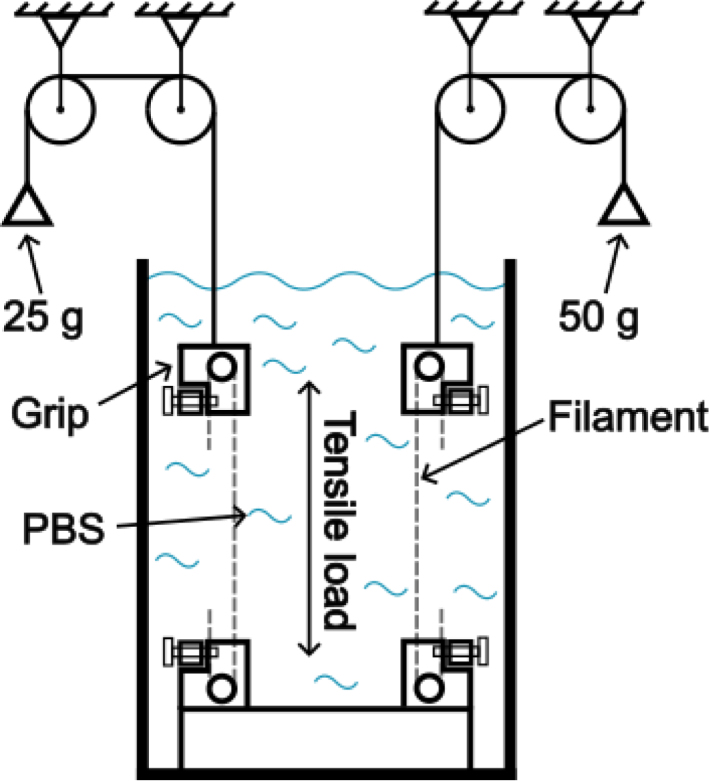
Schematic of the custom-designed degradation rig.

Degradation of the filaments under load was conducted in a custom-designed testing rig, consisting of an aluminium frame equipped with 3D-printed bollard grips made of thermoset resin (Formlabs, US), as shown in Fig. 2. Details about the design of the testing rig are provided in Appendix A. The frame was submerged in a 30 x 30 x 30 cm PBS tank, kept at constant temperature using silicone heating mats (RS Pro, UK) attached to its base and sides. The setup could accommodate up to 72 loaded ES PCL filaments simultaneously. In parallel, degradation studies on non-loaded samples were conducted using 50 mL test tube vials. Each filament was cut to a standardised length of 25 cm, and at least twelve ES filaments were degraded per time point, with six filaments placed in each vial. The vials were filled with PBS solution and then submerged in a shared water bath to ensure uniform thermal exposure throughout the testing period. The temperature and pH were continuously monitored using an Arduino Mega 2560 with a Ramps 1.4 board, equipped with a DS3231 real-time clock, three NTC temperature probes, a DF Robot pH sensor, and a micro-SD module for data logging. The Arduino also operated as a PID controller to regulate the heating mats and maintain precise water temperature. We have verified that the pH remained stable, averaging 7.5 ± 0.6 throughout the 5-week testing period.

Filaments were weighed before submersion, and in their wet and dry states following a predefined degradation period using a digital balance (Sartorius, Germany) with a 0.01 mg precision. After removal from the bath, wet samples were laid on a paper towel to remove excess water and weighed immediately, dried for 72 h at environmental temperature (i.e. 22 ± 2 °C) and re-weighed. The water uptake w and mass change f were determined as:

(1) $$w=\frac{m_{w}-m_{d}}{w_{d}},\;f=\frac{m_{i}-m_{d}}{m_{i}},$$

where $m_{i}$, $m_{w}$ and $m_{d}$ respectively represent the initial, wet and dry weights of the filaments. Due to the rapid drying of the filaments (approximately 10 min at room temperature), it was not possible to unmount and weigh the loaded specimens sufficiently fast to obtain accurate water uptake measurements for samples degraded under load. After weighing, the filaments were cleaned using an ultrasonic cleaning bath (Ultrawave, UK) to remove excess salt, dried, and stored for further characterisation.

### 2.4 Gel permeation chromatography

The molecular weight of the filaments was measured using an HPLC gel permeation chromatography (GPC) system (Shimadzu, UK). Samples of approximately 2.5 mg were dissolved in tetrahydrofuran (THF) solvent at a concentration of 10 mg ml^−1^. GPC analysis was conducted at an average flow rate of 1.0 mL min^−1^ at room temperature, with the system column calibrated using polystyrene standards of known molecular weights. The number average molecular weight $M_{n}$, weight average molecular weight $M_{w}$, and dispersity Đ $=M_{w}/M_{n}$ were calculated. Only one measure was taken for each degradation duration and load.

The evolution of number-average molecular weight with degradation time was fitted using two kinetic models from the literature. Both models are based on the assumption that the concentrations of ester bonds and water are constant during degradation, and neglect mass loss. The first model further neglects autocatalysis, giving (Pitt and Gu, 1987):

(2) $$\frac{1}{M_{n}}=\frac{1}{M_{n_{0}}}+k_{1}t$$

where $M_{n_{0}}$ is the initial number average molecular weight and $k_{1}$ is the rate constant. In contrast, the second model assumes that hydrolysis is autocatalysed by the acidic end groups produced by chain scission, leading to Pitt et al., 1981, Pitt and Gu, 1987:

(3) $$M_{n}=M_{n_{0}}\exp\left(-k_{2}t\right)$$

where $k_{2}$ is the rate constant in the model accounting for autocatalysis. Derivation details of these two models can be found for example in Lyu et al. (2007). Fitting of the experimental degradation curves was based on linear regressions of $1/M_{n}$ (Model 1) and $\ln\left(M_{n}\right)$ (Model 2) as a function of time, setting $M_{n_{0}}$ to the value for the undegraded polymer.

**Fig. 3:**
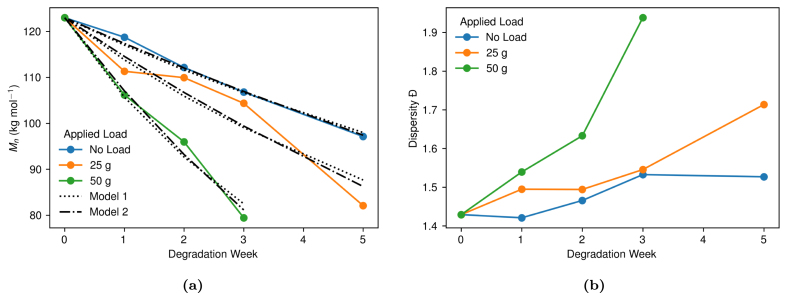
(a) Number-average molecular weight $M_{n}$ as a function of degradation time for specimens degrading with and without applied loads. Dashed lines are fits using two different models. (b) Dispersity Đas a function of degradation time.

### 2.5 Thermal properties

Differential scanning calorimetry (DSC) tests were conducted with a DSC Q2000 differential scanning calorimeter (TA Instruments, US) in a nitrogen atmosphere with a flow rate of 50 mL min^−1^ and aluminium hermetic cups with lid. Samples were first cooled to reach a thermal equilibrium of −90 °C, then heated at 10 °C min^−1^ up to 80 °C. Samples were then cooled at 10 °C min^−1^ down to −30 °C. The heat flow per unit mass (W g^−1^) was recorded to identify any phase transformations. DSC thermographs were analysed using TA Universal Analysis software. The enthalpy of fusion from solid to liquid ($\Delta H_{S\to L}$) and recrystallisation enthalpy ($\Delta H_{L\to S}$) were calculated from the areas under the respective peaks to determine the degree of crystallinity (K), using the following formula:

(4) $$K\left(\text{\%}\right)=\frac{\Delta H}{\Delta H_{0}}\times 100$$

where ΔH is the experimentally-measured melting enthalpy ($\Delta H_{S\to L}$ or $\Delta H_{L\to S}$), and $\Delta H_{0}$ is the reference value for 100% crystalline material, reported as 139.5 J g^−1^ (Pitt et al., 1981, Lam et al., 2008). Only one measurement was taken for each degradation duration and load.

### 2.6 Microstructural characterisation

The microstructure of the pre-stretched, undegraded specimens and dried, degraded specimens was examined using a TM3030Plus tabletop Scanning Electron Microscope (SEM, Hitashi, Japan). Specimens were mounted onto an aluminium stub using carbon adhesive disk. The SEM operated under variable pressure with a 15 kV acceleration voltage and 4.4–4.8 mm working distance. Micrographs were obtained from randomly selected regions at magnifications between x100 and x1000 to examine the mechanisms of degradation at both the fibre and filament scale. The filament and fibre apparent diameters were measured using ImageJ software. The reported average diameter values are based on micrographs corresponding to three different samples and at least eight recordings were taken for each sample.

### 2.7 Mechanical testing

The mechanical response of dried degraded ES PCL filaments was characterised using an Instron 5582 electromechanical tensile tester (Instron, UK) with a 100 N load cell. Filaments were cut to a standardised length of 120 mm as this was the minimum length required to wrap the filaments around the grips. Filaments were first wrapped around the top bollard and clamped. Wrapping around the lower component was carefully conducted to ensure vertical alignment and prevent any torsion of the filament. A 45 mm gauge length was defined by the two contact points at which the filament tangentially contact the grips. Mounting the filaments introduced some pre-load in the range 0.05–0.2 N. Uniaxial tensile tests were conducted at room temperature (22 ± 2 °C) and environmental humidity conditions (38 ± 5%).

Filaments were tested at an extension rate 75 mm min^−1^ until failure. This extension rate was selected for consistency with our previous work (Zanetti Ferreira et al., 2025), where we characterised the behaviour of the unstretched, undegraded filaments at three extension rates, 5, 75 and 150 mm min^−1^. The value of 75 mm min^−1^ was adopted in the present work as a representative value. The nominal strain was defined as ${ɛ}_{n}=\frac{L-L_{0}}{L_{0}}$, with $L_{0}$ and L the initial and deformed gauge lengths. The nominal stress was obtained by dividing the force by the cross-section area of the filament calculated from the average diameter determined by SEM and assuming a circular cross section (see also Section 3.3). For the filaments degraded without applied loads, the diameter of the undegraded, pre-stretched filaments was used ($d_{0}=467.08$ μm). For the filaments degraded under applied loads, the average diameters (in μm) calculated from SEM images were 359.84 ± 13.24, 335.22 ± 10.87, 302.73 ± 17.11, and 314.09 ± 35.7 after 1, 2, 3 and 5 weeks of degradation under 25 g load, and 294.92 ± 19.72, 286.05 ± 24.19 and 256.36 ± 19.85 after 1, 2 and 3 weeks of degradation under 50 g load. The apparent elastic modulus was calculated by fitting a linear regression in the linear elastic regime of the nominal stress–strain response. At least four samples were tested for each degradation condition. Statistical analysis was performed in Python using statsmodels and pingouin packages. Pairwise comparisons between independent groups with unequal sample sizes were evaluated using two-way ANOVA and the Games–Howell tests. Results were considered significant at p≤ 0.05.

## 3 Results

### 3.1 Degradation tests

Fig. 3(a) shows the evolution of $M_{n}$ with degradation time, for specimens degraded with and without applied loads. In the initial, undegraded state, the molecular weight is $M_{n}=123$ kg mol^−1^ with Đ= 1.42. The molecular weight then decreases with time over the considered time period. Despite the relatively short degradation period, the reduction in molecular weight is significant. This contrasts with previous findings by Chung et al. (2012), who did not observe any noticeable reduction in molecular weight in ES PCL sheets degraded in PBS at the same temperature for up to 42 days. Fig. 3(a) also shows that the molecular weight decreases faster under applied loads. For degradation without load, the molecular weight shows a 21.1% reduction by Week 5. In comparison, samples subjected to a 25 g load show a reduction of 33.2% after 5 weeks, and samples subjected to 50 g load show a molecular weight reduction of 35.4% after only 3 weeks (these samples broke before reaching 5 weeks of degradation). The evolution of the dispersity is shown in Fig. 3(b). Under no load, Đshows minimal variation during degradation, remaining between 1.42 and 1.55 over the study period. In contrast, the dispersity under a 25 g load increases from 1.42 to 1.72 by Week 5, and the dispersity under a 50 g load increases to 1.95 by Week 3.

Fig. 3(a) also shows the fitting of the experimental curves using Eqs (2), (3), respectively. Fitted experimental constants and $R^{2}$ values are provided in Table 1. Overall, both models fit the experimental reasonably well over the considered time period, with and without applied loads. However, the autocatalytic model provides a slightly better fit. A linear decrease of $\ln\left(M_{n}\right)$ with time was previously reported by Pitt et al., 1981, Pitt and Gu, 1987 in both *in vitro* and *in vivo* degradation of PCL at 37 °C. Other studies have reported similar degradation kinetics at 37 °C and neutral pH, e.g. Tsuji and Ikada, 1998, Zhang et al., 2013, Larrañaga et al., 2014, Fernández et al., 2015.

The water uptake during degradation of specimens degraded without load is reported in Table 2, showing an increase in water uptake with degradation time. The mass change of the filaments after a given degradation period and for each applied load is also reported in Table 2, showing an increase in weight. This is attributed to the deposition of salt from the PBS solution, which was observed in SEM images for unwashed filaments (images not shown). Similar mass increase was previously reported during degradation of porous PCL membrane (Gaona et al., 2012). The mass change appears to increase with the applied load. However, no consistent pattern is observed across different degradation periods.

**Table 1:** Rate constants of model 1 (Eq. (2)) and Model 2 (Eq. (3)) fitted on the experimental data.

	$k_{1}$ (mol kg^−1^ weeks^−1^)	$R^{2}\left(\text{\%}\right)$ (Model 1)	$k_{2}$ (weeks^−1^)	$R^{2}\left(\text{\%}\right)$ (Model 2)
0 g	$4.15\times 10^{-4}$	99.0	0.0468	99.6
25 g	$6.55\times 10^{-4}$	91.0	0.0728	93.1
50 g	$1.33\times 10^{-3}$	98.0	0.1386	98.9

**Table 2:** Water uptake and mass change of degraded filaments. The results are the average reported values using at least six filaments for the mass change and only two for water uptake.

	*Water uptake,*w (%)	*Mass change,*f (%)		
	No Load	No Load	25 g	50 g
Week 1	68.1	34.6 ± 22.6	52.7 ± 32.3	65.7 ± 53.9
Week 2	137.1	18.1 ± 5.1	105.7 ± 57.5	183.9 ± 123.1
Week 3	172.2	5.1 ± 10.3	72.0 ± 54.4	91.9 ± 62.2
Week 5	226.2	11.49 ± 22.1	258.6 ± 119.8	–

### 3.2 Thermal properties

**Fig. 4:**
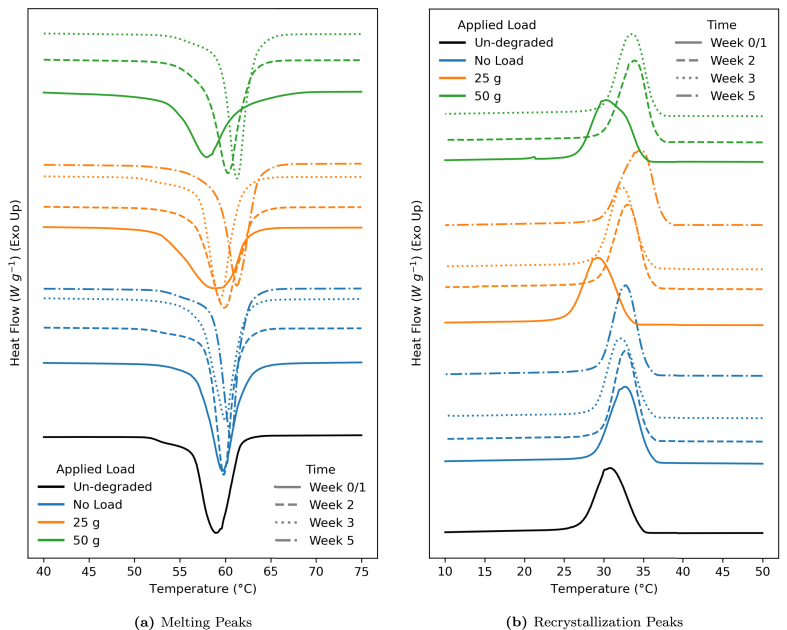
DSC thermographs of electrospun PCL filaments after different exposure time periods and applied loads, showing (a) melting peaks during heating and (b) recrystallisation peaks during cooling.

The DSC thermograph of an undegraded ES PCL filament is shown as the black-solid line in Fig. 4. Melting starts at 54.3 °C, with a peak at 59.0 °C (Fig. 4(a)), followed by a recrystallisation peak during cooling at approximately 30.7 °C (Fig. 4(b)). Fig. 4 also shows DSC thermographs for the filaments degraded under non-loaded and loaded conditions. Compared to the undegraded case, the endothermic (melting) and exothermic (recrystallisation) peaks show an increase in height, reduction in width, and slight shift of their positions to higher temperature with increasing degradation time. Mechanical loads applied during degradation appear to reduce the peak height.

**Fig. 5:**
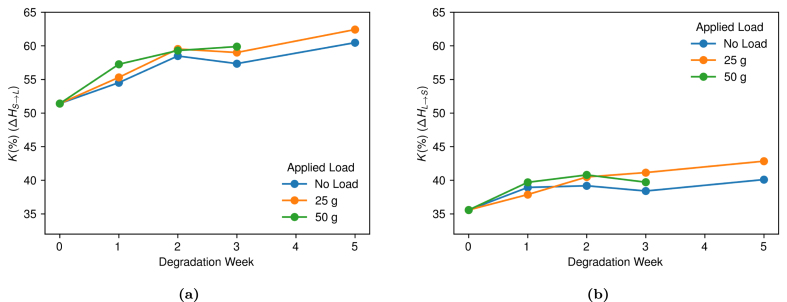
Degree of crystallinity calculated based on (a) $\Delta H_{S\to L}$ and (b) $\Delta H_{L\to }S$ in samples pre-degraded with and without applied loads.

The evolution of the degree of crystallinity with degradation time calculated based on $\Delta H_{S\to L}$ in Eq. (4) is shown in Fig. 5(a). The degree of crystallinity is 51.4% in the initial, undegraded state, and increases with both during exposure time and the applied load. The crystallinity of samples degrading under no applied load increases by approximately 18% between Week 0 and Week 5, whereas the degree of crystallinity of samples subjected to a 25 g load shows a 22% increase over the same time period. Samples degrading under a 50 g load show a 17% increase in crystallinity within just three weeks. Fig. 5(b) shows the degree of crystallinity of pre-degraded samples following a heating and cooling cycle, calculated based on $\Delta H_{L\to S}$. Undegraded samples reach a degree of crystallinity of about 35.6% when cooled from the melt, which is significantly lower than the initial degree of crystallinity. The crystallinity degree increases with the degradation time, and is enhanced in samples degraded under load.

**Fig. 6:**
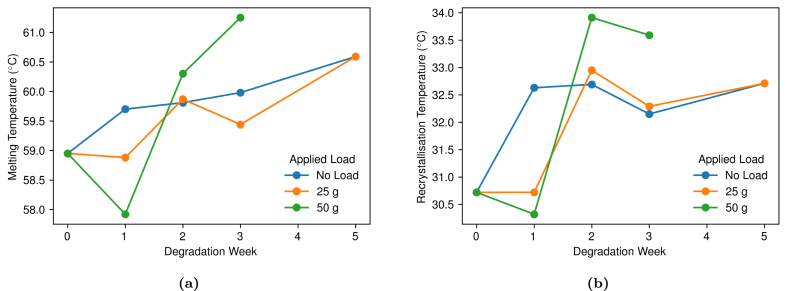
Evolution of (a) melting temperature and (b) recrystallisation temperature as a function of degradation time and for different applied loads.

Fig. 6 shows the evolution of the melting and recrystallisation temperatures as a function of degradation time, for different applied loads. For a given applied load, both temperatures increase overall with the degradation time, whereas the effect of the applied load for a given degradation period is less clear.

### 3.3 Microstructural characterisation

**Fig. 7:**
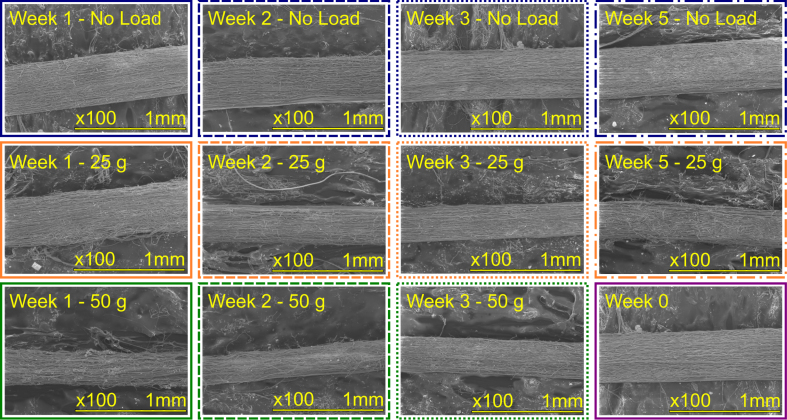
Representative SEM micrographs for each degradation time and applied load.

Fig. 7 shows SEM micrographs of filaments subjected to varying degradation durations and applied loads. Across all samples, the ES morphology is characterised by an anisotropic, porous, and densely packed fibrous network. For the same exposure time, the apparent diameter of the filament decreases with the applied load. The filament diameter also decreases with the exposure time for filaments degraded under load. Additionally, filaments subjected to a 50 g load show early signs of structural deterioration by localised fibre rupture.

**Fig. 8:**
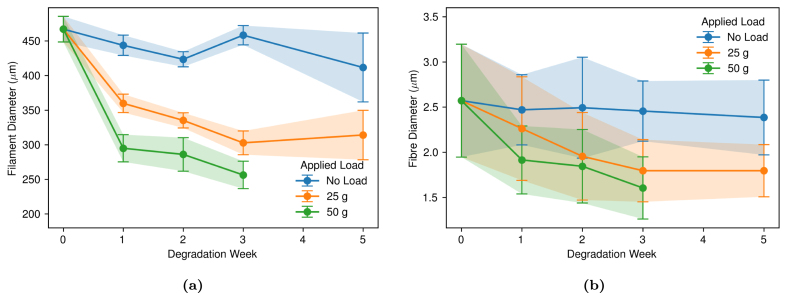
Evolution of the (a) filament and (b) fibre diameter with degradation time and for different applied loads. Diameters were calculated based on SEM micrographs. Shaded area represents a linear interpolation between the standard deviation for each averaged value.

Filament and fibre diameters measured across all degradation conditions are shown in Fig. 8 as a function of the degradation time. Numerical data are reported in Table 3. It should be noted that electrospun filaments are not perfectly cylindrical; their cross-sections are often flattened due to the electrospinning collection and stretching processes. Measurements correspond more to the width of the flatter side of the filament rather than a true diameter of a circular cross-section. For samples degraded under no applied load, the apparent filament and fibre diameters remain relatively constant over time, with variations falling within the bounds of standard deviation. In contrast, samples subjected to applied loads during degradation exhibit a progressive decrease in filament and fibre diameter during degradation, with a larger applied load causing a larger decrease in diameter for a given exposure time. The reduction in filament diameter indicates the occurrence of creep deformation during degradation under load, involving axial elongation of the filaments along with lateral contraction. The concurrent reduction in fibre diameter further indicates that filament creep involves fibre creep as well. These observations are consistent with our previous observations that stretching of ES filaments causes a reduction in both filament and fibre diameters (Zanetti Ferreira et al., 2025).

**Table 3:** Filament and fibre diameter.

	Filament diameter (μm)			Fibre diameter (μm)		
	0g	25g	50g	0 g	25 g	50 g
Week 0	467.08 ± 18.54	–	–	2.57 ± 0.62	–	–
Week 1	443.68 ± 14.64	359.84 ± 13.24	294.92 ± 19.72	2.60 ± 0.39	2.26 ± 0.57	1.92 ± 0.38
Week 2	423.52 ± 10.85	335.22 ± 10.87	286.05 ± 24.19	2.49 ± 0.56	1.96 ± 0.48	1.85 ± 0.41
Week 3	458.35 ± 13.97	302.73 ± 17.11	256.36 ± 19.85	2.40 ± 0.30	1.80 ± 0.34	1.61 ± 0.34
Week 5	411.62 ± 49.72	314.09 ± 35.71	–	2.39 ± 0.41	1.79 ± 0.29	–

### 3.4 Mechanical properties

**Fig. 9:**
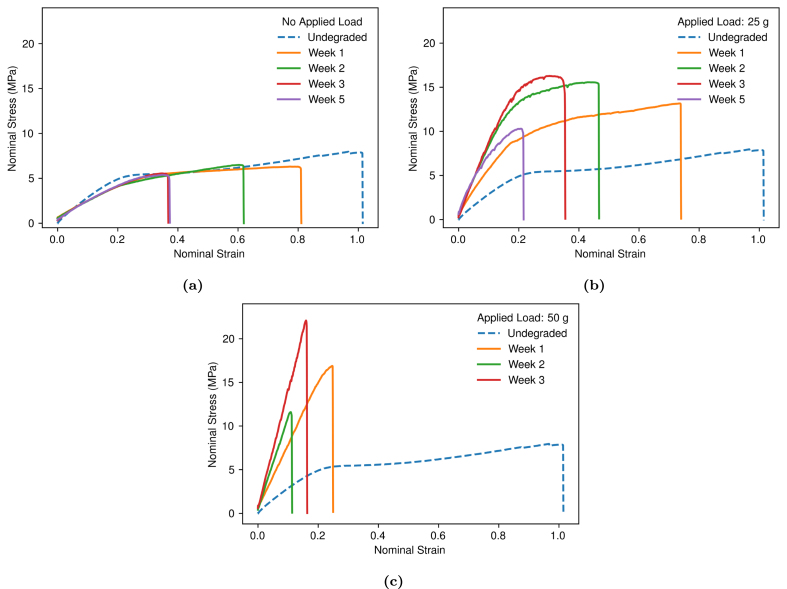
Representative stress–strain curves of filaments degraded under (a) no load, (b) 25 g, and (c) 50 g applied load. The extension rate was 75 mm min^−1^.

**Fig. 10:**
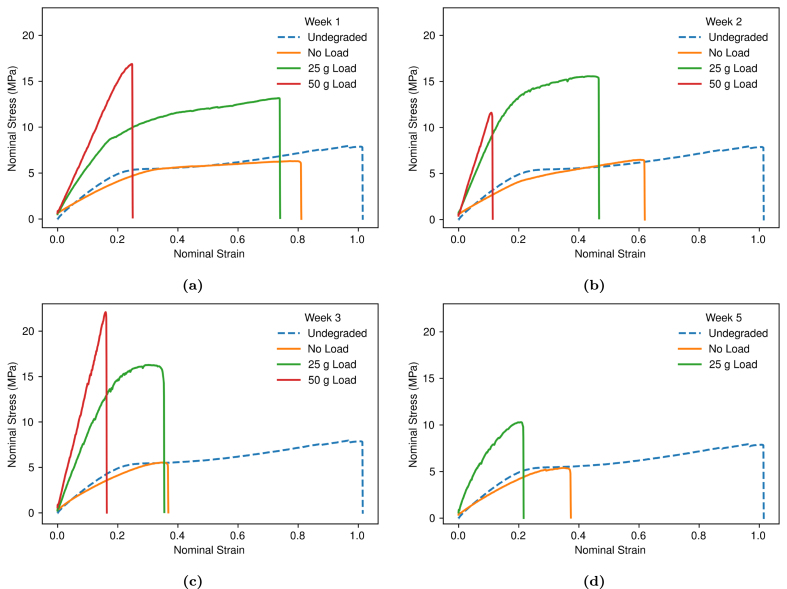
Representative stress–strain curves of filaments degraded for (a) 1 week, (b) 2 weeks, (c) 3 weeks and (d) 5 weeks and for different applied loads. The extension rate was 75 mm min^−1^.

Representative stress–strain curves of filaments degraded under no applied load are shown in Fig. 9(a) (refer to Appendix B for complete dataset). The response of the undegraded specimen is characterised by an initial linear elastic regime, followed by plastic deformation with mild strain hardening, until failure. The average apparent yield stress of the undegraded specimens is 4.7±0.95MPa, where the yield stress was measured using the 0.2% offset strain. A 50 g load corresponds to a 2.7 MPa stress based on a filament diameter of 467.1μm (Table 3) so that the loads applied during degradation were lower than the yield stress of the undegraded filament. The response of the degraded samples also exhibits elasto-plastic characteristics, with a lower apparent elastic modulus, lower yield stress and reduced hardening capacity. The most significant effect of degradation is the reduction in plastic deformation (ductility), causing a major reduction in the strain at break. Compared to samples degraded under no load, filaments degraded under 25 g load show an increase in elastic modulus and apparent yield stress, but a reduced ductility resulting in a smaller strain at break. In particular, some specimens degraded for more than 2 weeks show brittle-like behaviour and fail before yielding. (Fig. 9(b)). Degradation under a 50 g load causes a further increase in elastic modulus with degradation time. However, all these specimens fail before the yield point in a brittle-like manner and the strain at overall decreases with the degradation time (Fig. 9(c)). Note that specimens broke in the bath before reaching 5 weeks of degradation, so that no data could be collected at this time point. Fig. 10 shows the same representative stress–strain curves at each time point, for all three loading conditions. At each time point, increasing the applied load corresponds to higher initial stiffness and a lower strain at break.

**Fig. 11:**
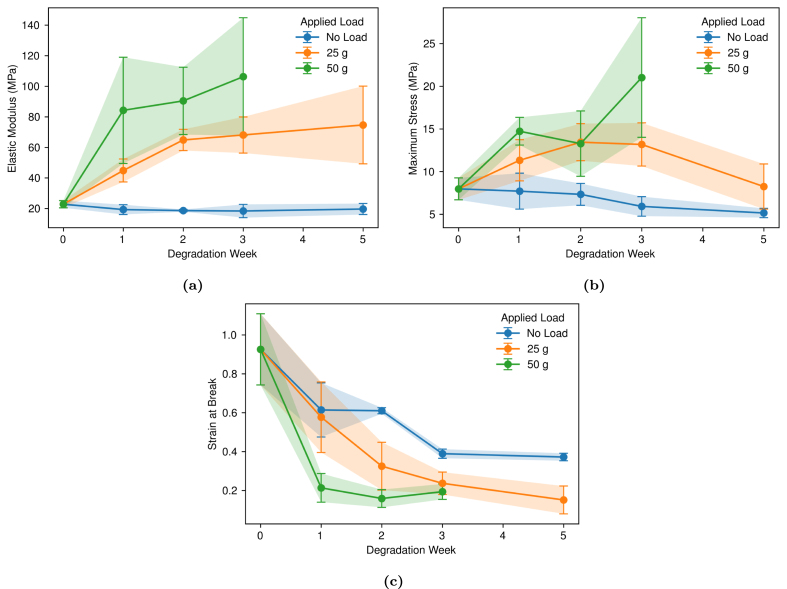
(a) Average elastic modulus (E), (b) maximum stress ($\sigma_{max}$) and (c) strain at break (${ɛ}_{break}$). At least 4 filaments were tested per each degradation condition. Shaded area represents a linear interpolation between the standard deviation for each averaged value.

The evolution of average elastic modulus is shown in Fig. 11(a), along with the standard deviation over four specimens. The undegraded samples have an apparent elastic modulus of 22.8 ± 2.4 MPa. Samples degraded under no applied load show a small reduction in elastic modulus after the first week of exposure, and the modulus appears to remain constant afterwards, at least during the considered degradation period. In contrast, samples degraded under applied load show an increase in apparent modulus with both degradation time and applied load. The evolution of elastic modulus with degradation time contrasts with the corresponding evolution of the overall degree of crystallinity (Fig. 5(a)), which show comparable increase in degrees of crystallinity with time for all three loading conditions, with a mild positive effect of the load. This suggests that the change in overall degree of crystallinity alone cannot explain the change in elastic modulus during degradation under load. The evolution of the maximum stress is shown in Fig. 11(b). While the maximum stress decreased with degradation time in non-loaded specimens, a non-monotonous evolution is observed in filaments degraded under a 25 g load, with an initial increase in maximum stress followed by a drop. In filaments degraded under a 50 g load, the maximum stress increases with exposure time overall. However, we recall no data point could be collected after 5 weeks. In contrast, the elongation at break decreases with exposure time in all loading conditions, and decreases as the applied load increases (Fig. 11(c)). For samples degraded under no load, a 40% reduction in strain at break is observed by Week 1, while for samples under 50 g load, the reduction reaches approximately 80%.

Measured mechanical properties show substantial variation across specimens (Fig. 11). Variability in the mechanical response of undegraded and unstretched ES filaments has previously been reported (Zanetti Ferreira et al., 2025), reflecting inherent heterogeneity in the electrospinning process and manifesting particularly in the maximum stress and strain at break. Our earlier work also demonstrated that these filaments are prone to inhomogeneous axial deformation (i.e., necking). In the present study, additional differences may arise from post-processing filament drawing, which can alter the degree of pre-stretch and further increase specimen-to-specimen variability. Nevertheless, Fig. 11 indicates that the degradation process itself introduces further variability, especially in the measured elastic modulus, for which the undegraded specimens initially exhibited excellent reproducibility.

**Fig. 12:**
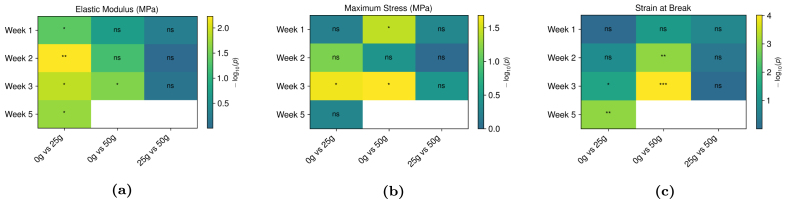
Games-Howell pairwise statistical comparison between applied loads at each degradation time point for (a) elastic modulus, (b) maximum stress, and (c) strain at break. Each cell indicates the significance level of each comparison: ns = not significant, *p<0.05, **p<0.01, ***p<0.001.

Games–Howell post-hoc tests were performed to assess the effect of loading conditions within each degradation time point for elastic modulus, maximum stress, and strain at break, with pairwise comparisons shown in Fig. 12. Significant differences were observed only at selected weeks. For the elastic modulus, differences were most consistently detected between unloaded and loaded filaments: no load versus 25 g was significant at all time points, while no load versus 50 g reached significance only in Week 3. No differences were found between 25 g and 50 g at any week. For maximum stress, significant effects were limited to Weeks 1 and 3, with Week 1 showing a difference between no load and 50 g, and Week 3 showing differences between no load and both 25 g and 50 g, while 25 g versus 50 g remained non-significant. For strain at break, no significant load-dependent differences were observed in Weeks 1 or 2, whereas Week 3 showed clear differences between no load and both loaded conditions, with again no significant contrasts between 25 g and 50 g across all weeks.

## 4 Discussion

### 4.1 Microstructure evolution during degradation

ES filaments considered in this study were subjected to a pre-stretch ratio of 7 prior to degradation. In our previous work (Zanetti Ferreira et al., 2025), we have characterised the mechanical behaviour and concurrent microstructure evolution of undegraded, unstretched filaments stretched using bollard grips for nominal strains up to about 11. The filaments were shown to exhibit a viscoelastic-viscoplastic response, with an initial modulus of 6.3 MPa and initial yield stress of 0.8 MPa at 75 mm s^−1^ extension rate, and an increase in hardening rate with the applied stretch. Deformation of the unstretched, undegraded ES filaments involved the progressive alignment of the fibres and the inelastic deformation of the individual fibres themselves, as evidenced by the reduction in fibre diameter measured by SEM at different stretching stages. We attribute the hardening response of the filament to the intrinsic response of the fibres as they align with the loading, involving molecular processes such as plasticity of the crystalline domains, alignment of the polymer chains in the amorphous regions and recrystallisation. In the present work, the filaments were pre-stretched to a nominal strain of approximately 7 before further testing, which is well into the plastic region of the unstretched filaments. As a result, the initial microstructure is already highly anisotropic, consisting of a densely packed network of ES fibres mainly aligned and pre-stretched in the loading direction (Fig. 7). As compared to the corresponding unstretched filament, pre-stretching thus increases the apparent modulus, apparent yield stress and hardening capacity.

**Fig. 13:**
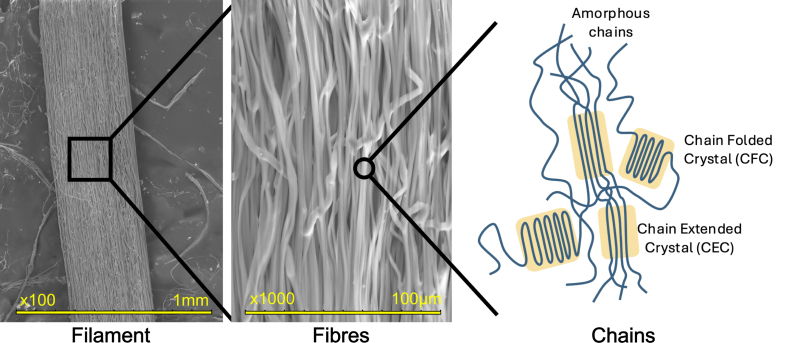
Schematic of the semi-crystalline microstructure of the PCL fibres, based on Chandler et al. (2025).

The microstructure of ES fibres of the same material was recently characterised by Chandler et al. (2025) using a combination of DSC, X-ray Diffraction (XRD) and Dynamic Mechanical Thermal Analysis (DMTA). These authors proposed that semi-crystalline fibres consist of chain-folded crystals (CFCs) and chain-extended crystals (CECs), whose relative proportions depend on the degree of pre-stretch. A high degree of pre-stretch would increase the proportion of CECs by unfolding of the CFCs and recrystallisation of extended chains in the amorphous domain and unfolded CFC. As a guide for our discussion, we assume in the following that the fibre microstructure of the pre-stretched filaments mainly consists of CEC crystals aligned in the stretching direction, and connected by the pre-extended chains in the amorphous phase (Fig. 13).

Based on Fig. 13, the mechanical response of the pre-stretched, undegraded filaments (blue curves in Fig. 9) can be interpreted as follows. The initially linear response is attributed to the elastic deformation of the crystalline domains and amorphous regions. The apparent plastic yielding may then be due to the fragmentation or unfolding of CFCs (as proposed by Chandler et al., 2025) and possibly the plastic deformation by crystallographic slip of the CECs. The moderate hardening could be attributed to the stretching of the chains in the amorphous regions, as they approach their extensibility limit, until failure by chain scission.

Hydrolytic degradation of PCL is generally considered to proceed by bulk erosion at body temperature and neutral pH conditions (Pitt et al., 1981, Bartnikowski et al., 2019). In particular, Bartnikowski et al. (2019) collected degradation data at physiological conditions from a large number of studies and found that the degradation rate was independent of the construct geometry, indicative of a bulk erosion process. In the present study at 45 °C, the highly porous structure of the ES filaments would promote the fast transport of water into the fibre structure, favouring bulk erosion. In addition, a significant reduction in molecular weight is observed while the filament and fibre diameters remain approximately constant in filaments degraded under no applied load (Fig. 8), which also supports the bulk erosion hypothesis, although some surface erosion cannot be entirely excluded. Although both degradation models fit the experimental data well, the superior fit of Model 2 suggests that the process may involve some degree of autocatalysis (Fig. 3(a)), consistent with previous findings in the literature (Pitt et al., 1981, Pitt and Gu, 1987).

The relatively rapid degradation observed in our study, compared to previous findings on PCL ES sheets by Chung et al. (2012), may be explained by the pre-stretching of the ES filaments. Bölgen et al. (2005) reported that reducing the filament diameter of ES PCL scaffolds increased their degradation rate, owing to enhanced water penetration and a higher surface-area-to-volume ratio. In our case, both pre-stretching and mechanical loading reduce filament diameter and increase the surface-area-to-volume ratio. Stretching of the filament may also promote partial uncoiling of polymer chains, exposing more amorphous regions and facilitating water accessibility to hydrolysable sites along the chains, thereby accelerating the degradation process.

Our DSC results show that degradation is accompanied by an increase in degree of crystallinity from approximately 50% in the pre-stretched, undegraded state to 60% after 5 weeks of exposure (Fig. 5(a)). The increase in crystallinity is more rapid during the first two weeks of degradation, which has been previously attributed to annealing of the polymer in the presence of water, enabling the growth of crystalline domaine (Pitt et al., 1981, Lam et al., 2008). The subsequent, slower increase in degree of crystallinity after two weeks could be attributed to a further increase in the chain mobility brought about by degradation (Little et al., 2009). Fig. 5(a) also suggests that applied tensile loads during degradation enhance recrystallisation, which can be attributed to strain-induced alignment and packing of the polymer chains, with CEC serving as nucleation sites for incorporation of amorphous chains (Chandler et al., 2025).

Overall, the melting temperature increases with degradation time (Fig. 6(a)), which is indicative of recrystallisation into more thermodynamically stable domains (rather than the loss of amorphous phase) (Tsuji and Ikada, 1998, Gaona et al., 2012). This is consistent with an increase in proportion of CEC crystalline domains (Chandler et al., 2025). However, the evolution of melting temperature with degradation under applied loads is non-monotonic, showing an initial decrease after one week of exposure (Fig. 6(a)). The effect is particularly significant for degradation under 50 g load, where the melting temperature drops after one week, before sharply increasing afterwards. It is possible that applied loads initially disrupt the existing crystalline domains, for example by breaking or unfolding of the CFCs, leading to the formation of a less stable and more heterogeneous crystalline structure. Over time, due to a combination of annealing and degradation, mobile chains are able to recrystallise into more stable crystalline domains, as reflected by the sharp rise in both melting and recrystallisation temperatures. However, it is worth noting that only one DSC test was carried out per condition. Further repeats need to be carried out to support these hypotheses.

### 4.2 Effect of mechanical loads on degradation kinetics

A key finding of this study is that degradation of ES PCL filaments is significantly accelerated in the presence of tensile loads (Fig. 3(a)). The effect of tensile loads on degradation kinetics could be due to the following factors. First, mechanical loads applied during degradation promote uncoiling of the polymer chains within the fibres, which could enhance access of water to the reaction sites, as previously mentioned in relation to the relatively fast degradation kinetics observed in this study. In addition, the stretching of the polymer chains during degradation could reduce the activation barrier for hydrolysis, according to principles of mechanochemistry (Akbulatov and Boulatov, 2017, Dong et al., 2024). This effect is expected to be more significant during the degradation of pre-stretched filaments, where the applied loads are more efficiently transferred to the pre-stretched polymer chains in the (degradable) amorphous regions between crystalline domains. However, it is unclear whether polymer chains are sufficient stretched to induce changes in molecular configurations, such as bond stretching and bond angle opening, for a mechanism of force-assisted hydrolysis to be active.

**Fig. 14:**
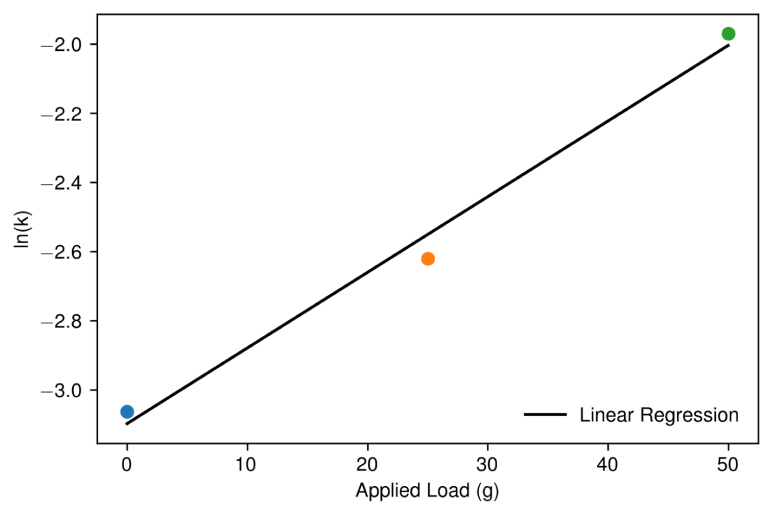
Dependence of the reaction rate constant $k_{2}$ on the applied load. The continuous line represents the fitting of experimental data with an Eyring-type equation.

Regardless of the actual physical mechanism(s) underpinning force-assisted degradation, the effect of applied loads on the reaction rate can be described phenomenologically using an Eyring-type equation (Kauzmann and Eyring, 1940, Bell, 1978):

(5) $$\;k_{\sigma }=k\exp\left(\frac{\alpha W}{k_{B}T}\right)$$

where $k_{\sigma }$ is the reaction rate in the presence of applied loads, k is the reaction rate in the absence of applied loads, $k_{B}$ is the Boltzmann constant, T is the absolute temperature, W is the applied load in grams, and α is an empirical parameter. The evolution of the rate constants of Model 2 are represented as a function of the applied load in Fig. 14, showing that they follow Eq. (5) with $\alpha =9.6\times 10^{-23}$ J g^−1^. Additional degradation studies involving a broader range of applied loads and temperature are however needed to determine the predictive capability of the empirical Eq. (5).

**Fig. A.15:**
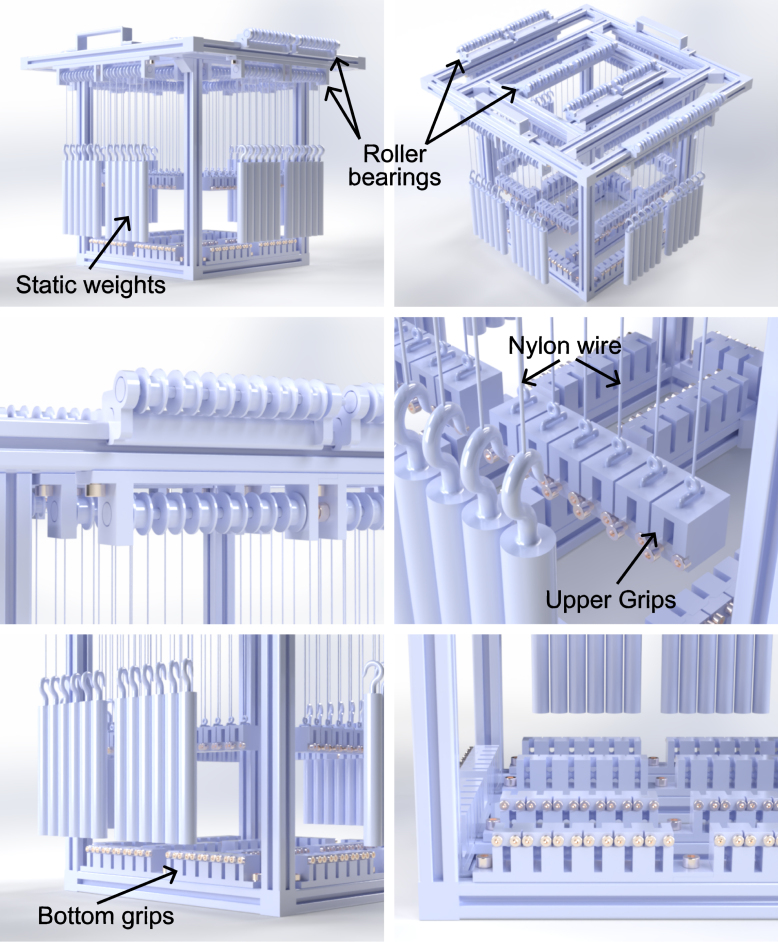
Computer-aided design (CAD) illustrations of the custom-designed degradation testing rig for ES filaments.

**Fig. B.16:**
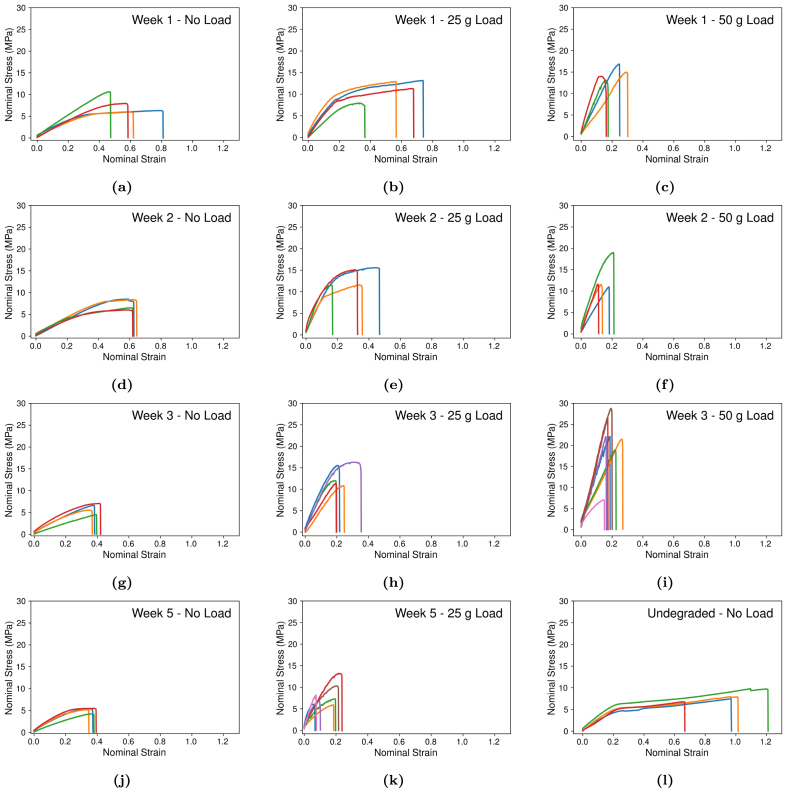
Complete dataset of all mechanical tensile tests conducted at an extension rate of 75 mm min^−1^. The first column corresponds to filaments degraded under no applied load, the second column corresponds to filaments degraded under a 25 g load, and the third column corresponds to filaments degraded under a 50 g load. Each row corresponds to a different degradation time point (Weeks 1–5). The response of undegraded filaments (Week 0) is shown in Fig. B.16(l).

### 4.3 Effect of degradation on mechanical properties

For specimens degraded without applied loads, the small initial drop in elastic modulus observed after one week of exposure 11(a) is likely due to the plasticisation effect of water, rather than hydrolytic degradation. For longer exposure times, a slight increase in modulus is observed, which could be the result of recrystallisation and associated change in proportion of CFC to CEC crystalline domains, balancing the effect of degradation in the amorphous phase. The reduction in strain and stress at break (Fig. 11(b) and 11(c)) with degradation time may be explained in the following way. As hydrolysis proceeds and long polymer chains are cut in the amorphous regions, the mechanical force transmitted along the fibre concentrates over a smaller number of chains, causing high stretch and premature failure of highly loaded chains by chain scission.

Specimens degrading under an applied load show a clear increase in elastic modulus with the exposure time. We attribute this effect to creep-induced microstructural changes during degradation under load. While the applied loads are smaller than the filament yield stress at room temperature, these loads are sufficient to cause significant creep deformation during degradation at elevated temperature. This can be seen from the reduction in filament diameter in Fig. 8(a), which suggests that the fibres continue to align in the loading direction during degradation under load, causing a further reduction in the filament porosity. Likewise, the reduction in fibre diameter shown in Fig. 8(b) (see also Table 3) indicates that the fibres themselves undergo significant creep deformations during degradation under load. For example, fibres in specimens degraded under a 25 g load for 5 weeks exhibit an average fibre stretch of about 2, as calculated from the reduction in fibre diameters and assuming incompressibility. We hypothesise that fibre creep involves the alignment and stretching of the polymer chains between the amorphous regions, increasing the fibre stiffness. Applied loads could also change the nature of crystallinity, with CECs enabling a more efficient load transfer from extended amorphous chains to the crystalline domains. On the other hand, the increase in overall degree of crystallinity during degradation (Fig. 5(a)) cannot explain the increase in modulus in specimens degraded under load, since it is similar for all three considered loading conditions, and therefore cannot explain the difference in apparent elastic modulus at a given degradation time point (Fig. 11(a)). An increase in CEC fraction could also explain the increase in yield stress, since CECs have a higher resistance to flow. The reduced strain at break and ductile–brittle transition during degradation under load (Fig. 11(c)) could be due to forces concentrating highly-stretched chains between the crystalline domains, causing chain scission. These microstructural changes could themselves be accelerated by force-assisted hydrolytic degradation.

## 5 Conclusion

This work investigated the degradation behaviour of ES PCL filaments, with particular attention to the interplay between hydrolytic degradation and applied loads. In the absence of applied load, a significant reduction in molecular weight is observed after 5 weeks of degradation in PBS at 45 °C, which translates into a reduction in mechanical properties. Tensile loads applied during degradation accelerate the reduction in molecular weight, which is primarily attributed to the uncoiling of the polymer chains under load, facilitating access of water to the reaction sites. Degradation under loads also leads to an increase in elastic modulus and the maximum stress, and to a significant decrease in the elongation at break. These effects are attributed to changes in the semi-crystalline morphology during degradation enabled by the increased mobility of the chains in the amorphous regions. It is also likely that the coupled effects of stress and degradation are amplified by filament pre-stretching after manufacturing. However, the underlying fundamental mechanisms could also be relevant to other polymers and manufacturing processes.

Overall, our findings highlight the need to consider mechanical loads in degradation studies, which impact both the reaction kinetics and the evolution of mechanical properties themselves. Such studies would enable more reliable predictions of mechanical performance and lifetime, which are critical for the design of biomedical implants in load-bearing applications. Future studies are needed to consider cyclic loading conditions over longer exposure times. Degradation studies at other (lower) temperatures are also needed to characterise the temperature-dependence of the rate constants, necessary for the extrapolation of accelerated degradation tests. Mechanistic models with predictive capability are also needed to guide the design of these materials.

## CRediT authorship contribution statement

**Thales Zanetti Ferreira:** Writing – original draft, Software, Methodology, Investigation, Formal analysis. **Huanming Chen:** Writing – review & editing, Investigation, Formal analysis. **Kaili Chen:** Investigation, Methodology, Writing – review & editing. **Pierre-Alexis Mouthuy:** Writing – review & editing, Supervision, Resources, Methodology, Funding acquisition, Formal analysis, Conceptualization. **Laurence Brassart:** Writing – review & editing, Writing – original draft, Supervision, Methodology, Funding acquisition, Formal analysis, Conceptualization.

## Declaration of competing interest

The authors declare that they have no known competing financial interests or personal relationships that could have appeared to influence the work reported in this paper.

## Data Availability

Data will be made available on request.

## Appendix A Experimental rig

A custom-designed degradation testing rig was developed to simultaneously apply static loads to a large number of ES filaments during degradation in aqueous media. The rig was designed to prevent mechanical interference between samples and maintain central alignment of the loading. Additional motivations included ease of assembly, cost-efficiency through 3D printing, and adaptability for various loading conditions.

Computer-aided design (CAD) illustrations of the rig are shown in Fig. A.15. The rig features a compact layout (the rig base measures 28 x 28 cm) and includes four peripheral walls and two additional internal pillars. Four vertical pillars, each 36 cm in length, connect the base to the top plate providing ample vertical clearance for each filament elongation within the water bath and weight suspension. The top section mirrors the base in layout but also incorporates an extended outer frame to house the pulley system.

The pulley system transmits load from the static weights to the upper grips via a two-point rolling element setup. This system uses nylon sewing thread, selected for its flexibility, mechanical strength, and resistance to aqueous degradation. The two-point configuration ensures vertical alignment of both the grips and weights, while integrated roller bearings minimise friction and ensure consistent force application throughout the degradation process.

Each side of the rig supports 12 filaments, using 3D-printed, bollard-style grips that were fabricated in two parts due to size limitations of the stereolithography printer. These grips provide secure filament fixation, maintain central loading alignment, and prevent tangling or twisting during testing. Combined with two centrally located support beams, the rig accommodates up to 72 filaments simultaneously. The central beams utilise an overhead pulley system, which spatially separates the weights, avoiding interference between pulley systems.

Mounting the filaments involved the following steps. ES filaments were cut to a standard length of 250 mm and mounted to the bottom bollard-style fixed grips. The free end of each filament was then attached to the unrestricted upper grip. The nylon sewing wire, was carefully looped around the roller-bearings system to prevent inducing any pre-stretching or damage to the filaments. Finally, a 25 g or 50 g weight hook was attached to an open-loop knot at the end of the nylon thread to apply the desired static load.

## Appendix B Stress–strain curves

Stress–strain curves of undegraded and degraded filaments for each degradation condition and degradation time are presented in Fig. B.16, including all repeats.
